# Older Adults’ Needs and Wishes for Contact With the Outdoors at Residential Care Facilities: Implications for Theory and Practice

**DOI:** 10.1177/19375867241276296

**Published:** 2024-10-24

**Authors:** Madeleine Liljegren, Anna Bengtsson, Göran Lindahl, Helle Wijk

**Affiliations:** 1 174416 Institute of Health and Care Sciences, University of Gothenburg, Gothenburg, Sweden; 2 Department of Architecture and Civil Engineering, 11248 Chalmers University of Technology, Gothenburg, Sweden; 3 Department of People and Society, The Swedish University of Agricultural Sciences, Alnarp, Sweden

**Keywords:** everyday environment, older adult, needs and wishes, outdoor environment, outdoor stay, residential care facility, walking interview

## Abstract

**Aim:** This qualitative study aimed to explore needs and wishes of older adults concerning their perceived need for contact with outdoor environments at residential care facilities (RCFs) and what implications it has for theory and practice. **Background:** There is increased awareness of the importance of health-promoting everyday environments for persons with special needs. Therefore, it is important to include the experiences of older adults at RCFs in research. **Methods:** Twelve older adults from three Swedish RCFs participated in semistructured walking interviews. **Results:** Two categories were identified concerning the needs and wishes of older adults for contact with outdoor environments. The first category, *Outdoor environments as part of everyday life*, describes aspects of normality linked to outdoor stays at RCFs. The second category, *Getting outdoors in practice*, describes supportive and hindering aspects of outdoor stays, as well as accessibility regarding different body positions and access to personal support. **Conclusion:** It was found in this study that the needs and wishes of older adults are important to consider to increase their opportunities for outdoor stays. Their needs and wishes could also be included in briefs and programs for the design and planning of new construction or refurbishment of RCFs. The results of the study can serve as the basis for further discussions concerning older adults’ outdoor stays and the accessibility of outdoor environments. Further, the results are intended to facilitate practical knowledge that is useful for care workers and managers at RCFs and to support decision makers, property developers, architects, and planners.

## Introduction

There is increased awareness of the importance of contact with nature and outdoor stays for health at all ages, especially for people with frail health (Bengtsson, 2015). A current challenge is the access to outdoor stays for older adults with disabilities living at residential care facilities (RCFs); this issue requires further research.

### Older Adults Living at RCFs

Being an older adult can include a reduced ability to cope with everyday life due to an aging-associated decline in bodily functions (Warmoth et al., 2016; Xue, 2011), such as frailty, cognitive decline, and decreased mobility (Ferrucci et al., 2016), which are common reasons for moving to RCFs (The Swedish National Board of Health and Welfare, 2011). RCFs are adapted housings for older adults who need special support around the clock from care workers and environment (Nordin, 2016; The Swedish National Board of Health and Welfare, 2011). Common professional groups at Swedish RCFs that support older adults are social workers who are assistant nurses and activity leaders as well as healthcare workers, such as nurses, occupational therapists, and physiotherapists (The Swedish Association of Local Authorities and Regions, 2023). Person-centered care and rehabilitation is a concept that unites professional groups in perceiving an older adult as a person with unique needs and wishes (Ebrahimi et al., 2021). Interventions such as meals, hygiene, dressing, movement, and activities exemplify the needs with which social care workers can support older adults. Healthcare workers’ support can consist of health talks, medication management, wound dressings, rehabilitation, and mobility aids (The Swedish National Board of Health and Welfare, 2023a). The physical environment should support the person-centered care and rehabilitation for example by offering private apartments adapted to the unique persons needs and dining areas for socialization and enough space to move with mobility aids (Nordin, 2016). RCFs in Sweden are mainly run by municipalities (approximately 80%), the rest (approximately 20%) are run by nonpublic providers (Broms et al., 2024).

### Health Effects of Contact With Outdoors

Outdoor views (Musselwhite, 2018) and outdoor stays contributes to contact with nature, which improve health and supports healing and recovery. The greatest health effects are evident for fragile and vulnerable persons (Ottosson & Grahn, 1998). The health effects of visiting gardens or participating in garden therapy can be measured in terms of decreased agitation, depression, need for medication, stress (Murroni et al., 2021), and risk of falling (Wolf et al., 2015). Outdoor stays lead to a higher degree of physical activity compared to indoor stays (Tenngart Ivarsson & Grahn, 2012) and counteract the negative consequences of inactivity (Cunningham et al., 2020), such as prolonged sitting (Douma et al., 2017). For persons with cognitive decline due to dementia, an adaptive and secure outdoor stay can offer the possibility of moving independently (Odzakovic et al., 2020) and choosing where one wants to be in an environment (Bengtsson, 2015). Ample greenery in the outdoor environments at RCFs appears to promote experiences of fascination and being away, leading to more frequent outdoor stays (Dahlkvist et al., 2016).

### Outdoor Mobility and Access to the Outdoors

Approximately one-third of older adults at Swedish RCFs experience deficient opportunities for outdoor stays despite these positive health-related outcomes (The Swedish National Board of Health and Welfare, 2023b). Mobility functioning includes the ability to move indoors and outdoors. It ranges from walking independently with or without support from care workers and/or mobility aids to being dependent and bedridden (Bengtsson, 2015). Previous research states that declining outdoor mobility is common among older adults (Skantz et al., 2020) and particularly for those who use mobility aids (Clarke, 2014). Moreover, aspects that affect autonomy can hinder outdoor stays. Such aspects could concern scheduled outdoor activities forcing older adults to adapt their lives to routines, understaffing, and employing temporary employees lacking the time to build relationships (Van Loon et al., 2021). A lack of convenience facilities outdoors (Kane, 2013), no easy access, or scarce cues to support orientation and wayfinding also can influence older adults’ ability to move between indoor and outdoor environments at RCFs and remain mobile outdoors (Bengtsson, 2015). Ensuring older adults’ access to outdoor environments is a priority on the international agenda, corroborating the UN's Sustainability Development Goal 11:7 (United Nations, 2022) and the World Health Organization's active work with the development of age-friendly cities and communities (World Health Organization, 2023). Thus far, the focus in previous research has been on how the environment supports preferences and outdoor usage (Rodiek et al., 2016), qualities of the environment and their relationship to wellbeing (Nordin, 2016), activities (Svensson et al., 2018), design aspects (Bengtsson, 2015), and accessibility (Iwarsson et al., 2012).

### Theoretical Framework

**Figure 1.**
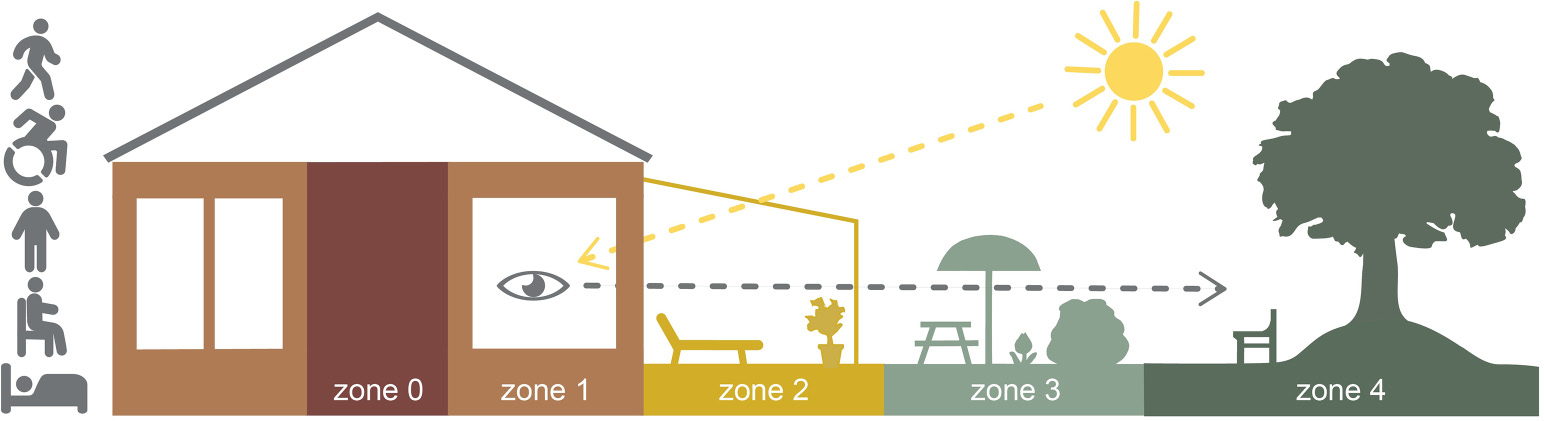
The principal model of four zones of contact with the outdoors. Illustration: A. Bengtsson.

According to Lawton's ecological model of aging (Lawton, 1983), environmental demands must be considered when designing RCFs for older adults to support positive affect and adaptive behavior. Combined with the principal model of the four zones of contact with the outdoors (Bengtsson, 2015), Lawton's model enables the exploration of artifacts in the outdoor environment that can influence the balance between older adults’ competence and environmental pressures. The principal model was developed within the research fields of landscape architecture and environmental psychology in healthcare. The model relates to use because it contributes a clear structure for different zones in the physical environment in which contact with the outdoors is possible. The first zone concerns contact with the outdoors from inside a building through its windows. The second zone concerns access to environments between the indoors and outdoors, such as balconies, patios, and conservatories. The third zone concerns access to the garden of the property, and the fourth zone is about having access to places in the surroundings. Zone 0 represents an indoor environment without contact with the outdoors. A concept of body position has been integrated into the model since persons in healthcare settings, due to functional capacities, are bound to certain body positions. Body positions that very much affect the possible contact with the outdoors and the possibility to move between the zones. The following body positions/functional capacities are included: (1) in motion (walking or in a wheelchair), (2) standing, (3) sitting, and (4) lying (Figure 1). The model emphasizes that all four zones should be present at RCFs and that it should be possible for older adults, regardless of their body position, to have contact with the outdoors, and to be able to move actively or passively in and between zones (Bengtsson et al., 2018). The bedridden may need to be moved in a wheeled care bed to an adapted place in zone 2 or 3. Both models highlight older adults’ need for adaptive environments to support their health and wellbeing. To summarize, we already know that needs for outdoor stays exist and that the design of the outdoor environments is important for health and wellbeing. However, there is a lack of knowledge that specifically focuses on supporting and hindering aspects in each zone and that also considers the different body positions. Therefore, there is a need of qualitative studies that complement the existing literature, to contribute to a more nuanced and fine-grained analytical perspective on older adults’ needs and wishes when using the different zones. To investigate and highlight this issue, the aim of this study was to explore needs and wishes of older adults concerning their perceived need for contact with outdoor environments at RCFs and what implications it has for theory and practice.

## Materials and Methods

This study takes an inter-disciplinary approach combining healthcare science, architecture, and landscape architecture. This work used a qualitative descriptive design based on semistructured interviews conducted in the form of walking interviews. A semistructured interview means that the interviewer starts from a number of overarching questions with the opportunity to adapt follow-up questions to deepen their understanding of the respondent's answers (Polit & Beck, 2021). A walking interview is a qualitative research method that is suitable for inter-disciplinary research focusing on the interaction between a person and the environment (King & Woodroffe, 2019). Walking interviews provide the possibility of understanding lived experiences in particular environments. To ensure the transparent and complete reporting of the research group and the study's design, analysis, and results, the authors followed consolidated criteria for reporting qualitative research, COREQ (Tong et al., 2007).

*Walking interviews provide the possibility of understanding lived experiences in particular environments.*

### Settings

**Figure 2.**
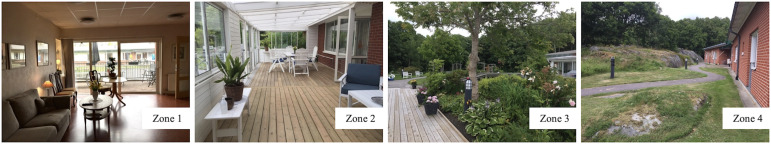
RCF 1: Zone 1–4. Photo: M. Liljegren.

**Figure 3.**
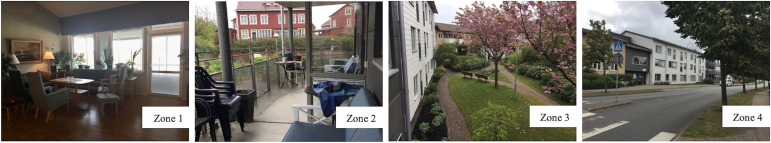
RCF 2: Zone 1–4. Photo: M. Liljegren.

**Figure 4.**
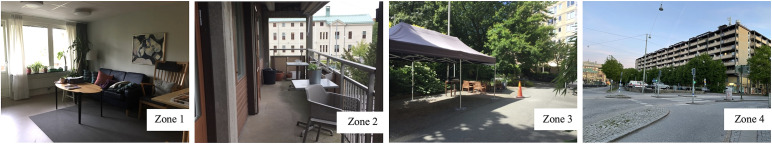
RCF 3: Zone 1–4. Photo: M. Liljegren.

Three RCFs in different-sized municipalities in Sweden were selected to achieve variations in contact and access to the four zones. Selection was based on municipal group classification according to the Swedish Association of Local Authorities and Regions (2023) in terms of population size, geographic density, and proximity to major cities and/or urban areas. Two RCFs had access to their own gardens, and the third had access to a shared courtyard park. RCF 1 was a one-floor building on the edge of a smaller community near a forest with access to outdoor environments such as shared patios, a conservatory, and a garden (Figure 2). RCF 2 was a three-floor building in the middle of a town with access to outdoor environments such as shared balconies, patios, and a garden (Figure 3). RCF 3 was a nine-floor building in a large city with access to balconies (both private and shared), a shared courtyard park, and proximity to a square (Figure 4). See Table 1 for a description of the significant parameters of the three RCFs.

**Table 1.** Matrix of Significant Parameters for the Three RCFs Included in the Study.

**RCFs**	**Municipal group classification**	**Location in the municipality**	**Number of floors in the building**	**Number of older adults at RCF**	**Zone 1**	**Zone 2**	**Zone 3**	**Zone 4**
**RCF 1**	Commuter municipality close to smaller city/town	Fringes of a town	1	35	Yes	Common patios and a conservatory	Large garden with vegetation and garden pond	Forest lot at the back
**RCF 2**	Commuter municipality close to a city	Middle of a town	3	71	Yes	Balconies and patios	Height variation in the backyard garden	Centrally located in a residential area
**RCF 3**	City	Central	9	84	Yes	Balconies	Shared garden/park	City, multistory buildings, traffic, classified as a particularly vulnerable area, proximity to squares and public transport

### Participants

The target group of the study was older adults living at RCFs. Purposive sampling was used to achieve diversity in older adults’ experiences based on variations in body position/functional capacity and health status. Four older adults were invited from each RCF: 12 persons from a total of 190 residents. The native language of all except one participant was Swedish. Those who used mobility aids used walkers or wheelchairs (both manual and electric), which means mobility aids with several wheels. A diagnosis of severe cognitive decline was an exclusion criterion. See Table 2 for a presentation of participants’ ages, body positions/functional capacity, years living at RCFs, and residential floors. Older adults living at the three RCFs received written information about the study. Those who wanted to participate were asked to report their interest to the managers who proposed selections for the research group based on the criteria above. On the same day that the interviews were conducted, the final inclusion was made based on actual health status and willingness. The participants gave their informed consent to the walking interviews: 12 older adults (nine women and three men) took part in individual interviews. Five older adults chose to bring a relative or care worker to their interviews for support. These support persons were asked not to engage actively in the interviews and were not included in the study.

**Table 2.** Description of the Participants (Ages, Body Positions/Functional Capacity, Years Living at RCFs, and Residential Floors).

**Ages**	**Number of older adults**	**Body positions/Functional capacity**	**Number of older adults**	**Years living at RCFs**	**Number of older adults**	**Residential floors**	**Number of older adults**
**71–80 years old**	2	In motion/standing independently	2	Less than 1 year	2	Floor 1	4
**81–90 years old**	4	In motion/standing, used walker independently	3	1 year	3	Floor 2	2
**91 years old or older**	6	In motion/sitting, used wheelchair independently	3	2 years	2	Floor 3	3
		In motion/sitting, used wheelchair, dependent on personal support	4	More than 2 years	5	Floor 4	1
						Floor 5	1
						Floor 6	1

### Data Collection

The semistructured walking interviews (King & Woodroffe, 2019) followed an interview guide with open-ended questions based on the principal model of the four zones of contact with the outdoors (Bengtsson, 2015). The questions concerned the participants’ experiences with each zone, the transitions between zones, and how they managed to use them. The interview guide was pilot tested on two older adults living at an RCF not included in the study. Subsequently, some linguistic adjustments were made. The walking interviews were conducted in summer 2022 and comprised visits in zones 1 to 3 and additional conversations about zone 4. The reason why zone 4 was not visited was due to the older adults’ fragile health and possible differences in time consumption depending on the distances to the places. Efforts were made to make the participants feel comfortable and safe and to adjust to the various interview situations. The walking interviews, which lasted between 28 and 80 min, were audio recorded and transcribed verbatim.

### Data Analysis

Qualitative content analysis (Lindgren et al., 2020) was used to systematize the content of interviews. Only manifest content was analyzed for a textual interpretation and an inductive approach (Graneheim et al., 2017). Data familiarization was undertaken by extensive reading and rereading of the entire dataset, followed by identifying meaning units and condensation, generating initial codes, and grouping them into subcategories and categories (see Table 3 for the number of meaning units, condensed meaning units, codes, subcategories, and categories). Further deliberations occurred within the research group to increase credibility and to reach a consensus (see Table 4 for the analytic process). The findings were reported in categories supported by quotations from the interviews. The quotations were abridged and modified to clarify their content before being translated into English.

**Table 3.** Number of Meaning Units, Condensed Meaning Units, Codes, Subcategories, and Categories.

**Meaning units**	**Condensed meaning units**	**Codes**	**Subcategories**	**Categories**
**1,455**	1,455	159	8	2

**Table 4.** Examples of Meaning Units, Condensed Meaning Units, Codes, Subcategories, and Categories.

**Meaning units**	**Condensed meaning units**	**Codes**	**Subcategories**	**Categories**
**I would like to be outdoors a little bit more.**	Be outdoors more	Wishes for more outdoor stays	Outdoor stays as a link between life in ordinary residences and at RCFs	Outdoor environments as part of everyday life
**Yes, for the threshold. It is very difficult to get outdoors (on one's own balcony).**	It is difficult to get outdoors because of threshold	The threshold between the indoors and outdoors hinders movement	Hindering aspects	Getting outdoors in practice

### Ethics

Participation in the study was voluntary. The walking interviews were conducted according to the Declaration of Helsinki (The World Medical Association, 2013), and ethical approval was obtained from the Swedish Ethical Review Authority (diary number: 2020-06643). Eleven older adults gave their written informed consent, and one gave oral consent due to the personal principle of not signing documents.

## Results

Two categories and eight subcategories illustrate the varied content relating to older adults’ needs and wishes for contact with outdoor environments at RCFs. The two categories are *Outdoor environments as part of everyday life* and *Getting outdoors in practice.* The categories and subcategories are described below; an overview is presented in Table 5.

**Table 5.** Categories and Subcategories.

**Categories**	**Subcategories**
**Outdoor environments as part of everyday life**	Outdoor stays as a link between life in ordinary residences and at RCFs
	Places for socializing and privacy
	Places for exercise, activity, and rest
	Outdoor stays despite the season and the weather
**Getting outdoors in practice**	Supporting aspects
	Hindering aspects
	Accessibility in relation to body positions
	Access to personal support

### Outdoor Environments as Part of Everyday Life

This category concerns aspects of normality, such as outdoor stays as a link between life in the older adults’ former ordinary residence and at the RCFs, and as a place for socializing, privacy, exercise, activity, and rest, despite the season and the weather.

#### Outdoor Stays as a Link Between Life in Ordinary Residences and at RCFs

This subcategory refers to the outdoor environment at RCFs functioning as a mechanism to recognize the former life in one's own residence and one's ongoing life at RCFs, a common aspect among older adults regardless of their body position. The possibility of moving between indoor and outdoor environments when desired enhanced this linkage, as did the use of flowers and berries in the RCFs’ gardens, which were common in residents’ former gardens. This recognition also strengthened the feeling of “coming home” to the RCFs after visits to the surroundings. The previously frequent outdoor stays in the former ordinary residence were described as wonderful, unlike the isolation indoors when this was no longer possible due to disabilities. The outdoor stays were described as still important for everyday life after moving to RCFs due to the importance of being able to leave the indoors whenever one wished. Older adults who could move independently between indoor and outdoor environments used this opportunity as much as they wanted and considered it a privilege. Dependent older adults wished for more (preferably daily) outdoor stays, and independent older adults recognized the lack of support for dependent older adults as a drawback. In fact, not being able to go outdoors to the desired extent was one of the things that was missed the most after moving to RCFs. “It's this (outdoor stays) I miss the most (after moving to an RCF).”—Woman, 91 years old or older (in motion/sitting, used wheelchair, and dependent on personal support).

*“It's this (outdoor stays) I miss the most (after moving to an RCF).”—Woman, 91 years old or older (in motion/sitting, used wheelchair, and dependent on personal support).*

#### Places for Socializing and Privacy

This subcategory refers to important and appreciated outdoor places for socializing and privacy. When meeting with other persons, spontaneous conversations often arose. “*It is the most fun when there are a few of us who come outdoors and sit here.”—Woman, 81 to 90 years old (in motion/sitting, used wheelchair, and dependent on personal support).* This raised opportunities for residents to show the gardens to relatives and acquaintances and use them together. Meals and coffee were part of the socializing for most of the older adults, regardless of body position, and appeared in various ways at balconies, patios, conservatories, and barbecues in the evenings. Eating outdoors was perceived as fun and cozy, and residents longed for it more often. Visiting balconies was described as positive and supported privacy, as well as walking in the surroundings. However, walking was also appreciated in company.

#### Places for Exercise, Activity, and Rest

Exercise, activity, and rest took place for all older adults in all four zones. Exercising was considered important for contributing to independence but was usually offered indoors, even though outdoor activities near nature were perceived as pleasant. Specifically on balconies in the fresh air, outdoor rest contributed to sleeping, regardless of older adults’ body positions. However, suitable furniture for sitting and lying down, pillows, and so on was missing. “*It (rest) is much cozier out here (on the balcony). … It's close to nature and close to the … outdoors.”—Woman, 91 years old or older (in motion/standing independently).*

##### Outdoor Stays Despite the Season and the Weather

Several older adults thought nice weather positively triggered outdoor stays year-round, but mostly during the spring and summer. Outdoor stays could even be conducted in rainy weather, although this occurred less often. “*You can be outdoors every day. If it rains, an umbrella can be used.”—Woman, 91 years old (in motion/standing and used walker independently).* Outdoor stays sometimes occurred during winter if snow was removed. Outdoor stays could still take place if heated conservatories were available despite the cold weather. Obstacles to being outdoors were strong winds and a lack of appropriate clothing and shoes. Even though residents were dressed for outdoor stays, support from care workers was still often needed due to weather conditions, particularly for those seated in wheelchairs.

### Getting Outdoors in Practice

This category refers to supportive and hindering aspects of outdoor stays. The category also includes results concerning accessibility regarding body positions and access to personal support.

#### Supporting Aspects

This subcategory describes the appreciated aspects supporting contact with the outdoors and outdoor stays. Larger windows entailed access to daylight and being able to lie in the care bed while maintaining contact with the outdoors. Trees provided support for some older adults when loneliness was experienced. Open windows or doors stimulated residents to move freely between the indoors and outdoors. Supportive aspects included electronic/automatic door openings and access to elevators for multiple floors. Such supportive aspects enhanced residents’ independence, particularly for those who used mobility aids regardless of body position. Other supportive aspects were access to balconies, patios, and conservatories, particularly for the opportunity to sunbathe regardless of body position and number of floors at RCFs. Having access to balconies in different directions entailed possibilities to choose between places with sun and shade. This freedom of choice also accounted for having access to several entrances, gardens with flowerbeds and ponds, and squares and parks in the surroundings. Additional supportive aspects were large patios, well-kept outdoor environments and balcony railings, which contributed to outdoor activities and reduced fall risks. Those with private balconies valued them; those who did not have balconies wanted them. “*It would have been nice to have your own balcony.”—Woman, 91 years old or older (in motion/standing independently).* Lack of supportive aspects in the environment was few places for group activities, such as boules courts. In Table 6, supporting aspects are presented in relation to zones 1 to 4 to provide an overview of aspects that influence the physical environments at RCFs.

**Table 6.** Overview of physical aspects supporting contact with the outdoor environment, and outdoor stays based on the results from this study.

Zone	Area	Supporting aspect
1	Indoor environment	Access to views contributed to contact with ongoing life outdoors Nature views contributed to support when experiencing loneliness Large windows provided daylight Openable windows (cleaned) and doors stimulated to outdoor stays Elevators in multi-floor buildings contributed to easy movement to the outdoor environment on ground level
	Transition through entrance	Electronic/automatic door opening facilitated movements when using mobility aids
2	Entrance room	Entrance doors with a possibility to choose between going outside to surroundings versus into the garden Furniture to sit on
	Balcony, patio, and conservatory	Easy access to private/shared/glazed balconies, balconies in different directions, and balconies for smoker/non-smoker Balcony railing Easy access to patios, and conservatories Large places for several older adults to use at the same time for socialization, activities, and meals Even floor surface
3	Garden	Attractive gardens (flower beds, bushes, trees, berries, and pond) Furniture for sunbathing Asphalt or other hard and even surfaces contributed to easy accessibility, especially when using mobility aids Partial enclosure by buildings contributed to an experience of oasis in the corner
4	Surrounding	Interesting features to watch Proximity to community services, squares, cafes, restaurants, parks, and relatives/acquaintances Proximity to nature elements Proximity to forest

#### Hindering Aspects

Several aspects hindered contact with the outdoors and made outdoor stays more difficult. Notable were long distances between one's apartment and the outdoors, too-small glazed balconies cramped by fellow residents, and high balcony railings that obstructed outdoor stays and views and contributed to disputes between residents. Tricky locking systems on doors and loose thresholds or boards increased feelings of being locked out and risking falling. “*Yes, once, right where you walk out (there was a fall accident in the transition between the conservatory/garden). It was a board that was crooked, and I fell and scraped my knee a bit.”—Woman, 81 to 90 years old (in motion/standing and used walker independently).* Additional hindrances were a lack of introduction and identification of outdoor places, which exacerbated the uncertainty about the gardens’ contents. Additionally, not knowing how to open the front door or how to find one's way back to the buildings exemplified hinders to outdoor stays. Another obstacle occurred when residents with and without cognitive decline had to share gardens, which could result in older adults without rarely using them due to the lack of conditions for conversation. Some older adults on an RCF's ground floor with access to openable French doors experienced the lack of a patio as a hindrance. Another complication for some older adults at RCFs in multifloor buildings was the perceived discomfort associated with elevator use. Those who lived at RCFs without access to nearby community services lacked the ability to visit a shop, for example. Additional hindrances mentioned by several older adults were visiting the surroundings, finding one's way back to the RCFs, and difficulties with distances between community functions. Dirty windows, messy balconies, dysfunctional garden ponds, and outdoor litter affected the willingness to invite relatives and acquaintances for visits. In Table 7, hindering aspects are presented in relation to zones 1 to 4 to provide an overview of aspects that influence the physical environments at RCFs.

**Table 7.** Overview of physical aspects hindering contact with the outdoor environment, and outdoor stays based on the results from this study.

Zone	Area	Hindering aspect
1	Indoor environment	Small windows contributed to a sense of being trapped Thresholds hindered when using mobility aids with wheels Doors opening manually was difficult when using mobility aids Elevator use contributed to discomfort
	Transition through entrance	Too short open time for door with electronic/automatic opening contributed to door slamming on the mobility aids Thresholds Loose thresholds contributed to risk of fall accident Door opener tag which was easy to forget contributed to being locked out
	Transition to balcony, patio, and conservatory	Thresholds Loose thresholds Narrow doorways especially when mobility aids were used
	Transition to garden	Long distance from apartment
2	Entrance room	Uncertainty where the electronic/automatic door opening button was located
	Balcony, patio, and conservatory	Lack of enough balconies, patios, and conservatories as they often were occupied of other older adults Too small/narrow shared and private balconies for older adults using mobility aids (lack of space) Wooden decking (the joints) Handrails blocking the view Lack of enclosing higher plants and trees Lack of adapted furniture with good comfort for activities, meals, and rest Messy places and outdoor litter contributed to reluctance to invite relatives and acquaintances Lack of wind shields on open balcony
	Transition to garden	Wooden decking with loose boards
3	Garden	Too narrow paths Lack of clear markings between paths and grass contributed to mobility aids ended up outside the paths Gravel Slopes hindered movement with mobility aids Stairs were unusable for older adults who used mobility aids Lack of handrails Shared garden (older adults with and without cognitive decline) contributed to the fact that older adults without seldom used the place due to the lack of conditions for conversation Lack of places where several persons can participate in activities together Lack of adapted furniture for activities, meals, and rest Lack of exercise equipment Lack of way-finding cues
4	Surrounding	Moving with mobility aids over curbs contributed to pain Lack of way-finding cues Traffic-noise Noisy children at preschool Open shared garden contributed to visits from undesirable persons Lack of proximity to shop

#### Accessibility in Relation to Body Positions

The experience of accessibility was connected to whether mobility aids were used or not. Accessibility independent of mobility aids was perceived as satisfactory indoors and outdoors. This contrasted with the need for walkers or wheelchairs, which were often problematic regardless of whether one was indoors or outdoors. Thresholds in the doorframes were deplorable and caused wheels to get stuck. When using mobility aids, uncertainty arose about how to handle the equipment inside and outside the elevator. Several older adults mentioned that manually opening doors independently was a difficult task. Doors that opened electronically were described as often slammed shut on residents because they needed more time to pass through. At entrances, it was difficult to handle the door opener tag, the door opening button, the mobility aid, and the threshold simultaneously. Regardless of body position and number of floors, wishes for increased accessibility were expressed to be able to get outdoors independently. “*It wouldn’t have been bad to get outdoors by myself.”—Woman, 81 to 90 years old (in motion/sitting, used wheelchair, and dependent on personal support).* Cramped balconies, patios, and conservatories hindered to turn mobility aids. Surface conditions such as gravel, grass, and slopes were difficult when using wheeled mobility aids, caused bodily discomfort, and required care workers’ support to drive onward. Although wooden decks had hard surfaces, they were perceived as problematic due to their joints as were stairs and too narrow garden paths, where the wheels could get caught in the grass. In addition, curbs negatively affected accessibility and generated body shocks, particularly when using a wheelchair. Environmental hindrances in surroundings decreased visits to relatives or acquaintances.

#### Access to Personal Support

Personal support relates to a range of needs, from walking arm hooks to the need for total support when seated in wheelchairs, which could result in older adults experiencing ignorance or a bad attitude when asking for help to go outdoors from care workers who seemed bothered. “It is difficult to get help, constantly asking for help and help and help and help.”–Woman, 71 to 80 years old (in motion/sitting, used wheelchair, and dependent on personal support). Care workers’ experience and movement techniques with mobility aids affected residents’ overall experience with the outdoor stay. Notions varied regarding whether older adults, regardless of body position, were allowed to go outdoors themselves, needed to inform care workers beforehand, or depended on care workers being able to accompany them, which resulted in some older adults never having visited the gardens.

*“It is difficult to get help, constantly asking for help and help and help and help.”—Woman, 71 to 80 years old (in motion/sitting, used wheelchair, and dependent on personal support).*

## Discussion

This study delved into the needs and wishes for contact with the outdoor environments at RCFs. In this section the theoretical standpoints are discussed and concretized. Further, everyday contact with the outdoors and considerations for outdoor stays are discussed. This section will also provide takeaways for practitioners, and it ends with some methodological considerations.

The study elucidates how supportive environmental aspects reduce environmental demands, thus giving offering concrete understanding in relation to Lawton's ecological model of aging (Lawton, 1983). The results relate to the model of four zones in contact with the outdoors (Bengtsson, 2015) and demonstrate how the four zones and transitions between them can be made available and accessible for older adults to benefit from contact with nature and outdoor stays. The results illustrated that participants used and expressed needs and wishes for contact with the outdoors in all four zones, which corroborates research on the importance of adapted outdoor environments to promote older adults’ mobility (Narsakka et al., 2021). The model of four zones in contact with the outdoors supports the understanding of the results by targeting access to the zones and the transitions between them, depending on the body's position. In addition, the results describe supporting aspects facilitating movements within and between zones as well as hindrances to making outdoor environments attractive and part of everyday life.

### Everyday Environments in All Four Zones

The results contribute to existing research on the importance of outdoor environments at RCFs (Bengtsson, 2015; Iwarsson et al., 2012; Nordin, 2016; Rodiek et al., 2016; Svensson et al., 2018). According to Sjögren et al. (2022), everyday environments at RCFs should be therapeutic and include the physical environment, the person's doing and being there in, and the organizational philosophy of care. This corroborates this study's results and indicates the importance of the outdoors as part of the everyday environment of older adults, where all four zones must exist to fulfill their needs and wishes. Correspondingly, it is important to reflect on how older adults are affected if one or two of these zones do not exist. Therefore, access to zones must be ensured already at RCFs’ planning stages, especially in larger dense cities where densification is taking place and various actors are interested in the properties. Densification can result in a lack of access to a garden at ground level, that is, that zone 3 is missing. Compensatory roof terraces might be provided, but could be difficult for older adults to use due to lack of accessibility and experiences with outdoor stays on roofs. The absence of one's garden at ground level also means lacking the freedom to choose where one wants to be in the environment. Lacking opportunities for outdoor stays in gardens reduces wellbeing and disappoints older adults at RCFs (Dahlkvist et al., 2019). Another trend is that newly built RCFs are planned with zone 3 as courtyards, with buildings framing the garden. This reduces the risk of older adults with orientation difficulties getting lost outdoors. Consequently, this provides no possibility for important contact with zone 4 (Odzakovic et al., 2020). The needs and wishes for transitions between zones add to the existing literature on the conditions for moving between indoor and outdoor environments (Bengtsson, 2015; Nordin, 2016). Obstacles comprise of mainly level differences (thresholds) and tricky door-locking systems described previously (Nordin, 2016). For environments to be developed from hindering to supportive, new solutions must provide the same levels of surface in all transitions between zones and that there is no risk of being locked out. Each ward's common space should have an entrance to a windowed elevator that leads directly to an outdoor environment.

…*all four zones must exist to fulfill their needs and wishes*

### Considerations for Outdoor Stays at RCFs in the Future

The results indicate that older adults dependent on care workers, relatives, or acquaintances to move in and between zones did not get the same opportunity for outdoor stays as independent older adults. This finding underscores that their expressed wishes for preferably daily outdoor stays must be catered to. This result substantiates research stating a lack of accessibility to outdoor environments for persons needing mobility aids (Zhang et al., 2017). Other research depicts that older adults with the most fragile health experience the greatest positive health effects of outdoor stays (Ottosson & Grahn, 1998). Consequently, the persons who benefit the most from outdoor stays are those whose needs are not met today, according to this study. In addition, older adults at RCFs do not receive sufficient support for physical activities outdoors, such as walking (Cedervall, 2014). Research on outdoor stays that includes older adults and persons with long-term health issues recommends 120 min of nature exposure weekly as a guideline. It does not matter how time is distributed (White et al., 2019). It is important to offer outdoor stays regularly, regardless of the season and weather. This is further motivated by our findings that outdoor stays contribute to normality. However, it is also important to be aware that outdoor stays can lead to negative health consequences in terms of heat stress, which has been a problem for children in preschools (Wallenberg et al., 2023) and should be considered when planning outdoor environments as well for older adults.

*…older adults dependent on care workers, relatives, or acquaintances to move in and between zones did not get the same opportunity for outdoor stays as independent older adults.*

### Takeaway Messages for Practice

The knowledge herein is useful for care workers and managers at RCFs and for decision makers, property developers, architects, landscape architects, and facility planners. This knowledge can be used when developing operations for outdoor stays and when developing design of physical environments. Investing in the development of supportive outdoor environments for older adults aligns with the UN's Sustainability Development Goal 11:7 (United Nations, 2022). This knowledge is also useful for relatives and acquaintances because the older adults expressed wishes to show and use outdoor environments together with them. By adapting efforts to an individual's needs and wishes, opportunities are provided for social engagement and access to meaningful everyday life (Chenoweth et al., 2018; Ebrahimi et al., 2021).

### Methodological Considerations

The semistructured walking interview method (King & Woodroffe, 2019) worked well to generate useful data. The selection of municipalities, RCFs and participants contributed to intended variation of access to the four zones. A critical reflection regarding the study's results is that none of the older adults who participated in the interviews expressed negative opinions about outdoor stays, perhaps because only persons with positive attitudes agreed to participate in the data collection.

The data collection was done in a short time frame by the same two researchers (the first and second authors). This approach ensured that all data were conducted in similar ways, as advocated by Graneheim and Lundman (2004) and Lindgren et al. (2020). The interview guide was pilot tested to ensure credibility, and all participants and managers received the same information from the first author to achieve dependability. Interactions with environments during interviews contributed to rich data by giving the researchers opportunities to observe the use of the different zones and to ask follow-up questions. An advantage was that the method allowed individual interviews and could be adapted to each older adult's specific body position/functional capacity. A challenge with outdoor data collection was the windy weather, which the researchers managed by urging older adults to wear outer clothing. Another limitation concerned the interview questions. Consideration was given to the older adults’ individual capacity to participate in the interviews and go outdoors. Some of the more detailed questions were excluded for older adults with especially fragile health so the interviews would not be too long and tiring. Another limitation could be not involving the frailest older adults, who were mostly bedridden. There could be a lack of insight into the perspectives of older adults with a diagnosis of dementia and non-Swedish-speaking persons.

The data analysis was carried out by all four researchers. A challenge of the data's possibility of having more than one meaning can arise in the analytic phase, depending on the researcher's preunderstanding and degree of interpretation (Graneheim et al., 2017). Collective competence was used to avoid the risk of one researcher's viewpoints coloring the results. Credibility and transferability were also ensured because categories and subcategories were anchored in the data and illustrated with quotations. The results generated by the study were generally expected, which can be explained by the fact that the questions in the interview guide were pragmatic. Older adults with various body positions and various degree of dependency for movement in the environments were included, so the results obtained can be considered to provide a comprehensive understanding of the wide range of needs and wishes for contact with the outdoor environment at RCFs. The knowledge from this study can be applied to older adults at RCFs with a diagnosis of dementia. Such individuals also often have physical disabilities, which are mirrored by the body positions in this study. A strength of this study is that frail older adults have been involved in semistructured walking interviews. Thus, they have had the opportunity to make their voices heard in research.

## Conclusion

Older adults at RCFs have specific needs and wishes for their contact with outdoor environments, especially those who depend on care workers, relatives, and acquaintances. Increased knowledge of older adults can serve as a basis for planners to develop supportive outdoor environments at RCFs and as an inspiration for care workers and managers to develop outdoor stays to be an integrated part of everyday person-centred care and rehabilitation.

## Implications for Practice

- It is important for care workers, managers, relatives, and acquaintances to recognize the specific needs and wishes of older adults for contact with outdoor environments at RCFs, especially for those who use mobility aids and need personal support to move between indoor and outdoor environments as well as in outdoor environments.
- The outdoor environments at RCFs should be considered an everyday environment for older adults, which means that care workers and managers must develop their operations accordingly.
- Older adults’ experiences with supporting and hindering aspects can be utilized in design solutions for new construction or refurbishment of RCFs, this data is useful for property developers, architects, planners, and representatives of the healthcare sector.
- Older adults’ experiences regarding lack of outdoor stays at RCFs could prompt national and local decision makers to develop guidelines for care and rehabilitation that include the possibilities to outdoor stays.
